# Two-year outcomes after full-thickness astigmatic keratotomy combined with small-incision lenticule extraction for high astigmatism

**DOI:** 10.1186/s12886-020-01756-8

**Published:** 2021-01-09

**Authors:** Bu Ki Kim, Young Taek Chung

**Affiliations:** 1 Onnuri Smile Eye Clinic, Hyobong building 9F 1, Gangnam-daero 65 gil, Seocho-gu, Seoul Republic of Korea; 2 Onnuri Eye Hospital, 325, Baekje-daero, Wansan-gu, Jeonju-si, Jeollabuk-do Republic of Korea

**Keywords:** Astigmatism, Astigmatic keratotomy, Small incision lenticule extraction, SMILE, High astigmatism, Limbal relaxing incision, LRI

## Abstract

**Background:**

To evaluate clinical outcomes after full-thickness astigmatic keratotomy (FTAK) combined with small-incision lenticule extraction (SMILE) in eyes with high astigmatism.

**Methods:**

This study comprised 75 eyes of 43 patients with over 4.0 diopters (D) of astigmatism who were treated with SMILE after FTAK. Visual acuities and refractive measurements were evaluated at 1 month after FTAK, and 1, 6, 12, and 24 months after SMILE. Vector analysis of the astigmatic changes was performed using the Alpins method.

**Results:**

Twenty-four months after the combined procedure, the average spherical equivalent was reduced from − 6.56 ± 2.38 D to − 0.36 ± 0.42 D (*p* < 0.001). The uncorrected and corrected distance visual acuities improved from 1.54 ± 5.53 to − 0.02 ± 0.09 and from − 0.03 ± 0.07 D to − 0.07 ± 0.08 D (both *p* < 0.001), respectively. The preoperative mean astigmatism was − 5.48 ± 1.17 D, which was reduced to − 2.27 ± 0.97 D and − 0.34 ± 0.26 D at 1 month after FTAK and 24 months after SMILE, respectively (*p* < 0.001). The surgically-induced astigmatism after FTAK, SMILE, and FTAK and SMILE combined was 3.38 ± 1.18 D, 2.22 ± 0.84 D, and 5.39 ± 1.20 D, respectively. Furthermore, the correction index of FTAK, SMILE, and FTAK and SMILE combined was 0.63 ± 0.17, 0.90 ± 0.40, and 0.98 ± 0.06, respectively. There were no intraoperative or postoperative complications.

**Conclusion:**

Our surgical procedure combining FTAK and SMILE showed good and stable clinical outcomes during two-year follow-up for the treatment of high astigmatism.

## Background

Small-incision lenticule extraction (SMILE) is a flapless, all-femtosecond laser-based technique for correcting myopia and myopic astigmatism, which has gained widespread acceptance due to its good predictability, safety, and efficacy [1–3]. However, correction of high astigmatism with SMILE is still challenging. According to several studies, astigmatism remains 11 ~ 16% undercorrected after SMILE, with higher preoperative astigmatism increasing the amount of the astigmatic undercorrection [4–6]. And because astigmatic correction is also limited to 5.0 diopters (D), a large amount of astigmatic undercorrection is predicted to occur when treating an eye with more than 5.0 D astigmatism using SMILE alone.

Previously, we reported good short-term clinical outcomes after using a surgical procedure combining cc and SMILE for patients who were inoperable using SMILE alone due to high astigmatism [7]. However, the number of cases was small and the duration of follow-up was only 6 months. Furthermore, we only analyzed the magnitude of astigmatic correction by FTAK and SMILE did not perform vector analysis.

In the current study, we retrospectively analyzed the clinical outcomes after SMILE preceded by FTAK for eyes with over 4.0 D astigmatism. Furthermore, astigmatic changes were analyzed after each procedure individually and after the two of them using vector analysis.

## Methods

### Patients

This retrospective study recruited 75 eyes of 43 patients who were treated with SMILE after FTAK at the Onnuri Smile Eye Clinic, Seoul, Korea from October 2015 to February 2017. All eyes had preoperative astigmatism of 4.0 D or more, a stable refraction for at least 1 year, and no preoperative topographic sign of keratoconus. Other inclusion criteria were a minimum age of 18 years, a corrected distance visual acuity (CDVA) of 20/25 or better, the absence of ocular disease, and a follow-up period of at least 2 years after SMILE. This study was approved by the Public Internal Regulatory Board of the Ministry of Health and Welfare, Korea (P01–202003–21-011). All procedures conformed to the tenets of the Declaration of Helsinki, and written informed consent for study participation was obtained from all patients.

Preoperatively, patients underwent a complete ophthalmologic examination including uncorrected distance visual acuity (UDVA), CDVA, manifest and cycloplegic refractions, slit-lamp microscopy, dilated fundus examination, specular microscopy (noncom Robo-ca®; Konan Medical, Hyogo, Japan), and dual rotating Scheimpflug analyzer (Galilei®; Ziemer Ophthalmology, Port, Switzerland). Experienced optometrists performed all examinations.

### Full-thickness astigmatic keratotomy

After scrubbing the skin and eyelids with povidone-iodine, the eyes were anesthetized with 0.5% proparacaine HCL (Alcaine®, Alcon, Fort Worth, TX, USA), and an eyelid speculum was used to keep the eye open in the recumbent position. The steepest axis was marked with a surgical marking pen with the aid of Callisto Eye system (Carl Zeiss AG, Dublin, CA), and a ring was marked with a 7.5-mm diameter ring marker and gentian violet. A beveled full-thickness corneal incision was made using a 2.8-mm keratome at 0, 0.5, 1.0, or 1.5 mm from the posterior ring marking, and the incision tunnel was about 1.0 mm in length. The incision was widened using a wider keratome (4.1 or 5.7 mm) depending on the magnitude of astigmatism (Table 1). One or two astigmatic keratotomies were performed according to the magnitude of preoperative astigmatism. The same nomogram as that used in our previous study was applied [7]. After checking for leakage with a Weck-Cel sponge, a mixture of cefazolin, prednisolone, and lidocaine in a 1:1:1 ratio was injected subconjunctivally near the incision. After the procedure, patients were treated with 0.5% moxifloxacin and 0.1% fluorometholone for 4 weeks.

**Table 1:** Nomogram of the full-thickness astigmatic keratotomy

Distance from corneal marking (mm)	Incision width (mm)	Corrected astigmatism (D)
1.5	2.8	0.75
1.5	4.1	1.25
1.5	5.7	2.5
1.0	2.8	1
1.0	4.1	1.75
1.0	5.7	3
0.5	4.1	2
0.5	5.7	3.5
0	5.7	4.5

### Small-incision Lenticule extraction

SMILE was performed when the refraction had been stable for 2 weeks, at least 4 weeks after FTAK. Emmetropia was the target refraction for all eyes. The SMILE procedure was performed with VisuMax® femtosecond laser (Carl Zeiss Meditec AG, Jena, Germany). The following laser parameters were used: 500-kHz repetition rate, 140-nJ pulse energy, 4.0-μm spot spacing, 110 to 120-μm cap thickness, and 6.0 to 6.7-mm lenticule diameter according to the diameter of the scotopic pupil and manifest refraction. And an incision tunnel was located at the 11 o’clock position. The lenticule was separated with a blunt spatula using Chung’s swing technique, as described in detail previously [8]. After removing the lenticule, the stromal pocket was flushed with balanced salt solution (BSS; Alcon). Following the procedure, patients were treated with 0.5% moxifloxacin for 5 days, 0.1% fluorometholone for 4 weeks, and preservative-free hyaluronic acid lubricating drops for at least 4 weeks.

### Outcome assessment

Measurement of UDVA, CDVA, and manifest refraction was performed at 1 month after FTAK and 1, 6, 12, and 24 months after SMILE to assess visual and refractive outcomes. Slit-lamp examination and dual-rotating Scheimpflug analysis were performed at every follow-up to detect postoperative complications. To assess the changes in postoperative astigmatism, vector analyses were performed in this study using the Alpin’s method [8, 9]. In brief, refractive astigmatism at the spectacle plane was converted to the corneal plane using a vertex distance of 12 mm. Refractive astigmatism was then analyzed by taking into account the change in the astigmatic axis and measuring 3 vectors and the relationships among them. The three vectors were defined as follows: the target induced astigmatism (TIA) vector represented the astigmatic change that the surgery intended to induce; the surgically induced astigmatism (SIA) vector represented the astigmatic change that the surgery actually induced; and the difference vector (DV) represented the difference between TIA and SIA. When the target cylinder is emmetropia, DV is equal to the postoperative residual astigmatic vector. The magnitude of error (ME) is the arithmetic difference between the SIA and TIA in magnitude. The angle of error (AE) is half of the axis difference between SIA and TIA. Reported in degrees, AE values indicate a clockwise error of the axis when negative. The correction index (CI) was defined as the ratio of SIA to TIA. The value is ideally 1; values less than 1 represent an astigmatic undercorrection, while values larger than 1 represent an astigmatic overcorrection. The index of success (IS) was defined as DV divided by TIA. Preferably, the IS value should be 0. Vector analyses were performed at three different time points, namely preoperatively, 1 month after FTAK, and 24 months after SMILE. The vector parameters of FTAK, SMILE, and FTAK and SMILE combined were subsequently compared. The safety index (the ratio between postoperative CDVA and preoperative CDVA) in addition to the efficacy index (the ratio between postoperative UDVA and preoperative CDVA) were calculated.

### Statistical analysis

Graphs were made using Graphpad Prism (Prism version 8.1.0; Graphpad Inc., La Jolla, CA) and Microsoft Excel 2016 (Microsoft, Inc., Redmod, WA, USA). The data were analyzed with SPSS software (version 20.0; SPSS Inc., Chicago, IL). All values are given as means ± standard deviation. All statistical analyses of visual acuity used logarithms of the minimum angle of resolution (logMAR). Statistical differences with *p* < 0.05 were considered significant. One-way analysis of variance (ANOVA) was used to assess the time-course of changes in visual and refractive outcomes, as well as the vector parameters corresponding to the different surgical procedures. Correlation analyses were performed using Pearson’s method.

## Results

This study included 75 eyes from 43 patients. The refraction of all eyes was stable at 4 weeks after FTAK, and all eyes had SMILE surgery at 4 ~ 6 weeks after FTAK. The mean preoperative spherical equivalent (SE) was − 6.56 ± 2.38 D and the mean preoperative astigmatism was − 5.48 ± 1.17 D. Preoperative characteristics of the eyes are presented in Table 2.

**Table 2:** Preoperative patient characteristics

Parameter	Mean ± SD (range)
Age (years)	25.3 ± 5.59 (18 to 38)
Sex (% female)	57.33
LogMAR UDVA	1.54 ± 5.53 (0.8 to 2.0)
LogMAR CDVA	−0.03 ± 0.07 (− 0.2 to 0.2)
CCT (μm)	534.36 ± 32.53 (472 to 611)
Sphere (D)	−3.82 ± 2.56 (− 7.75 to 2.25)
Cylinder (D)	− 5.48 ± 1.17 (− 9.0 to − 4.0)
SE (D)	−6.56 ± 2.38 (− 10.875 to − 0.75)

*logMAR* logarithm of the minimum angle of resolution, *UDVA* uncorrected distance visual acuity, *CDVA* corrected distance visual acuity, *CCT* central corneal thickness, *D* diopters, *SE* spherical equivalent

### Visual and refractive results

**Fig. 1:**
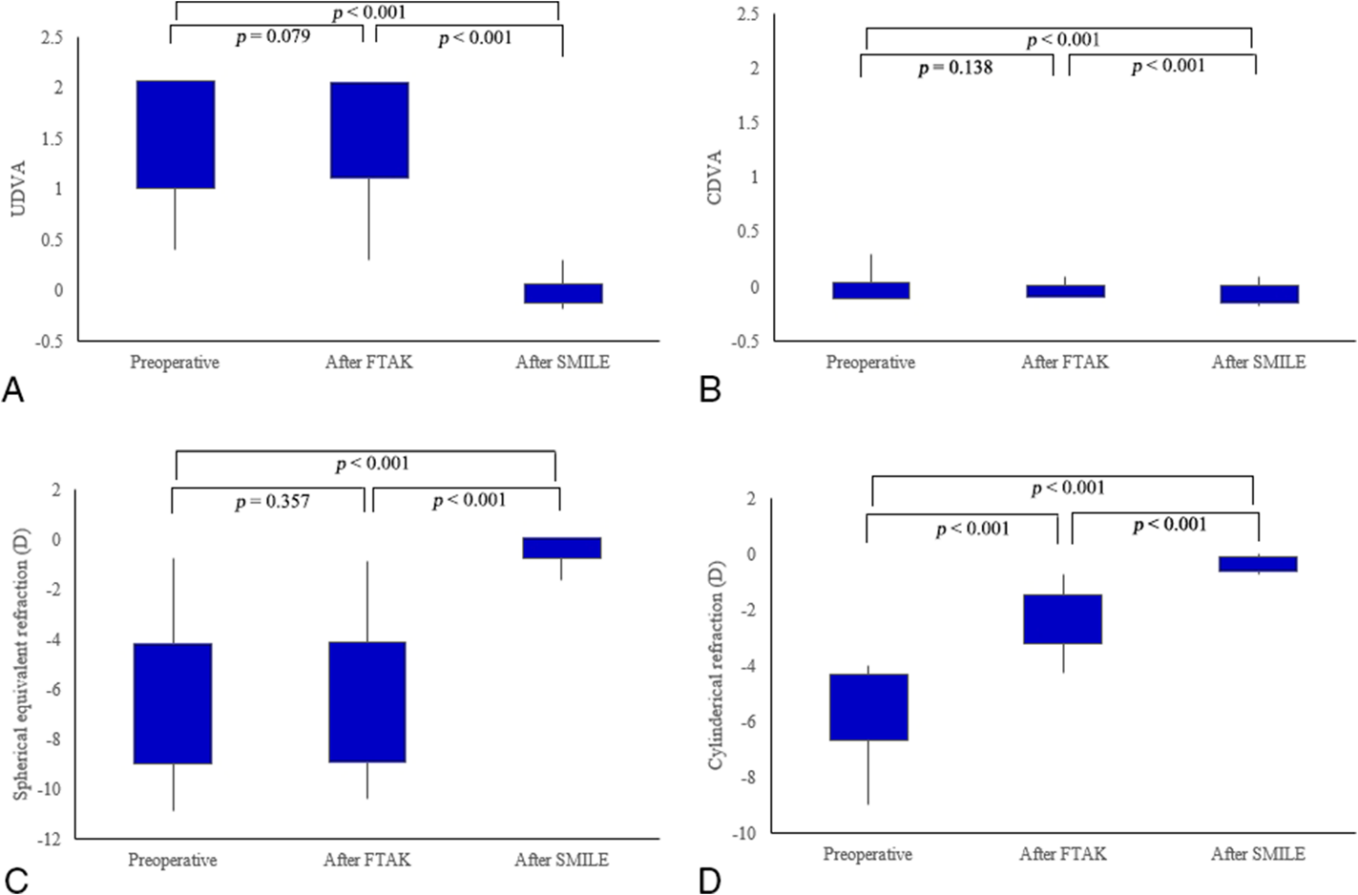
Visual acuities and refractive outcomes at 1 month after full-thickness astigmatic keratotomy (FTAK) and 24 months after small-incision lenticule extraction (SMILE). **a** Changes in uncorrected distance visual acuity. **b** Changes in corrected distance visual acuity. **c** Changes in spherical equivalent refraction. **d** Changes in cylindrical refraction

The mean UDVA was 1.54 ± 5.53 logMAR preoperatively, compared to 1.58 ± 0.47 logMAR at 1 month after FTAK (*p* = 0.079) and − 0.02 ± 0.09 logMAR at 24 months after SMILE (*p* < 0.001). The mean CDVA was − 0.03 ± 0.07 logMAR preoperatively, compared to − 0.04 ± 0.05 logMAR at 1 month after FTAK (*p* = 0.138) and − 0.07 ± 0.08 logMAR at 24 months after SMILE (*p* < 0.001). Figure 1 shows changes in the UDVA and the CDVA following the surgical procedures, with the combined procedures having a significant effect on both the UDVA and the CDVA (both *p* < 0.001). While no significant change in SE was observable 1 month after FTAK (*p* = 0.357), the astigmatism reduced from − 5.48 ± 1.17 D to − 2.31 ± 0.86 D, 1 month after FTAK (*p* < 0.001). Twenty-four months after SMILE, the mean SE was − 0.36 ± 0.42 D and the mean astigmatism was − 0.34 ± 0.26 D. The effects of the combined procedures on both SE and astigmatism were significant (both *p* < 0.001).

**Fig. 2:**
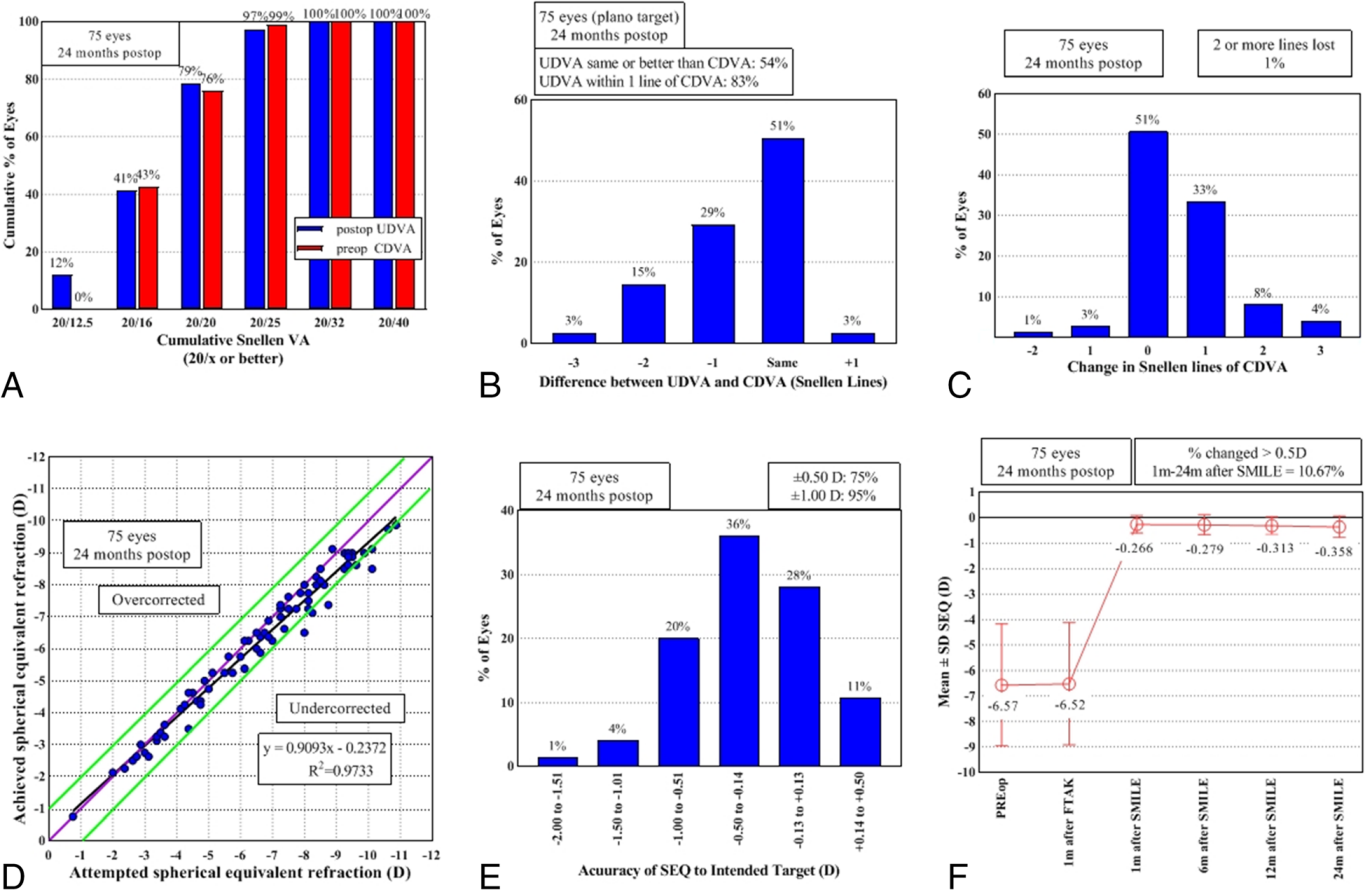
Refractive and visual outcomes after the combined full-thickness astigmatic keratotomy (FTAK) and small-incision lenticule extraction (SMILE) procedures

A UDVA of 20/20 or better was achieved by 79% of the eyes at 24 months postoperatively (Fig. 2a). The UDVA was 0.001 ± 0.07 logMAR at 1 month after SMILE and did not change during the postoperative period (*p* = 0.082, Table 3). Also at the 24-month follow-up, 75 and 95% of the eyes were within ±0.5 D and ± 1.0 D of attempted SE, respectively (Fig. 2e). Correlation between attempted versus achieved SE refraction was high (R^2^ = 0.9733, Fig. 2d). Moreover, 51% of the eyes had an unchanged CDVA, 4% lost one Snellen line or more, and 45% gained one Snellen line or more (Fig. 2c). The mean CDVA was − 0.05 ± 0.08 logMAR at 1 month after SMILE and did not change during the postoperative period (*p* = 0.272, Table 3). Overall, the mean SE was stable and did not show significant change throughout the postoperative period (*p* = 0.441, Table 3), with − 0.36 ± 0.42 D at 24 months (Fig. 1f). The efficacy and safety indices at 24 months after SMILE were 1.01 ± 0.24 and 1.10 ± 0.17, respectively. After the combined procedures, there was no change in the efficacy and safety indices during the follow-up period (Table 3).

**Table 3:** Visual acuities, refractive errors, efficacy index, and safety index

	Preoperative	1 month after FTAK	1 month after SMILE	6 months after SMILE	12 months after SMILE	24 months after SMILE	*p* value
LogMAR UDVA	1.54 ± 5.53 (0.8 to 2.0)	1.58 ± 0.47 (0.5 to 2.00)	0.001 ± 0.07 (− 0.1 to 0.1)	−0.03 ± 0.06 (− 0.2 to 0.1)	−0.03 ± 0.07 (− 0.2 to 0.1)	−0.02 ± 0.09 (− 0.2 to 0.1)	0.082
LogMAR CDVA	−0.03 ± 0.07 (− 0.2 to 0.2)	−0.04 ± 0.05 (− 0.2 to 0.2)	−0.05 ± 0.08 (− 0.2 to 0.1)	−0.07 ± 0.07 (− 0.2 to 0.1)	−0.06 ± 0.07 (− 0.2 to 0.1)	−0.07 ± 0.08 (− 0.2 to 0.1)	0.272
Sphere (D)	− 3.82 ± 2.56 (− 7.75 to + 2.25)	−5.37 ± 2.60 (− 9.25 to − 0.25)	−0.08 ± 0.30 (− 0.75 to 0.75)	−0.12 ± 0.37 (− 1.5 to 0.75)	−0.14 ± 0.35 (− 1.0 to 0.5)	−0.19 ± 0.41 (− 1.5 to 0.5)	0.382
Cylinder (D)	−5.48 ± 1.17 (− 9.0 to − 4.0)	− 2.27 ± 0.97 (− 4.25 to − 0.25)	−0.36 ± 0.28 (− 1.0 to 0)	−0.33 ± 0.26 (− 1.0 to 0)	−0.34 ± 0.29 (− 1.25 to 0)	−0.34 ± 0.26 (− 1.0 to 0)	0.946
SE (D)	−6.56 ± 2.38 (− 10.875 to − 0.75)	−6.52 ± 2.40 (− 10.375 to − 0.875)	−0.27 ± 0.33 (− 0.875 to 0.375)	−0.29 ± 0.38 (− 1.625 to 0.625)	−0.31 ± 0.34 (− 1.125 to 0.25)	−0.36 ± 0.42 (− 1.625 to 0.25)	0.441
Efficacy index	–	0.05 ± 0.08 (0.01 to 0.5)	0.94 ± 0.16 (0.58 to 1.43)	1.00 ± 0.19 (0.67 to 1.71)	1.01 ± 0.17 (0.67 to 1.5)	1.01 ± 0.24 (0.56 to 1.71)	0.130
Safety index	–	1.02 ± 0.10 (0.83 to 1.6)	1.06 ± 0.21 (0.58 to 1.71)	1.10 ± 0.17 (0.83 to 1.71)	1.09 ± 0.16 (0.83 to 1.43)	1.10 ± 0.17 (0.83 to 1.71)	0.292

*FTAK* full-thickness astigmatic keratotomy, *SMILE* small-incision lenticule extraction, *logMAR* logarithm of the minimum angle of resolution, *UDVA* uncorrected distance visual acuity, *CDVA* corrected distance visual acuity, *D* diopters, *SE* spherical equivalent. Values are presented as mean ± standard deviation (range)

*p* value of postoperative measurement (after SMILE), with one-way ANOVA

### Outcomes of astigmatism correction and vector analysis

**Fig. 3:**
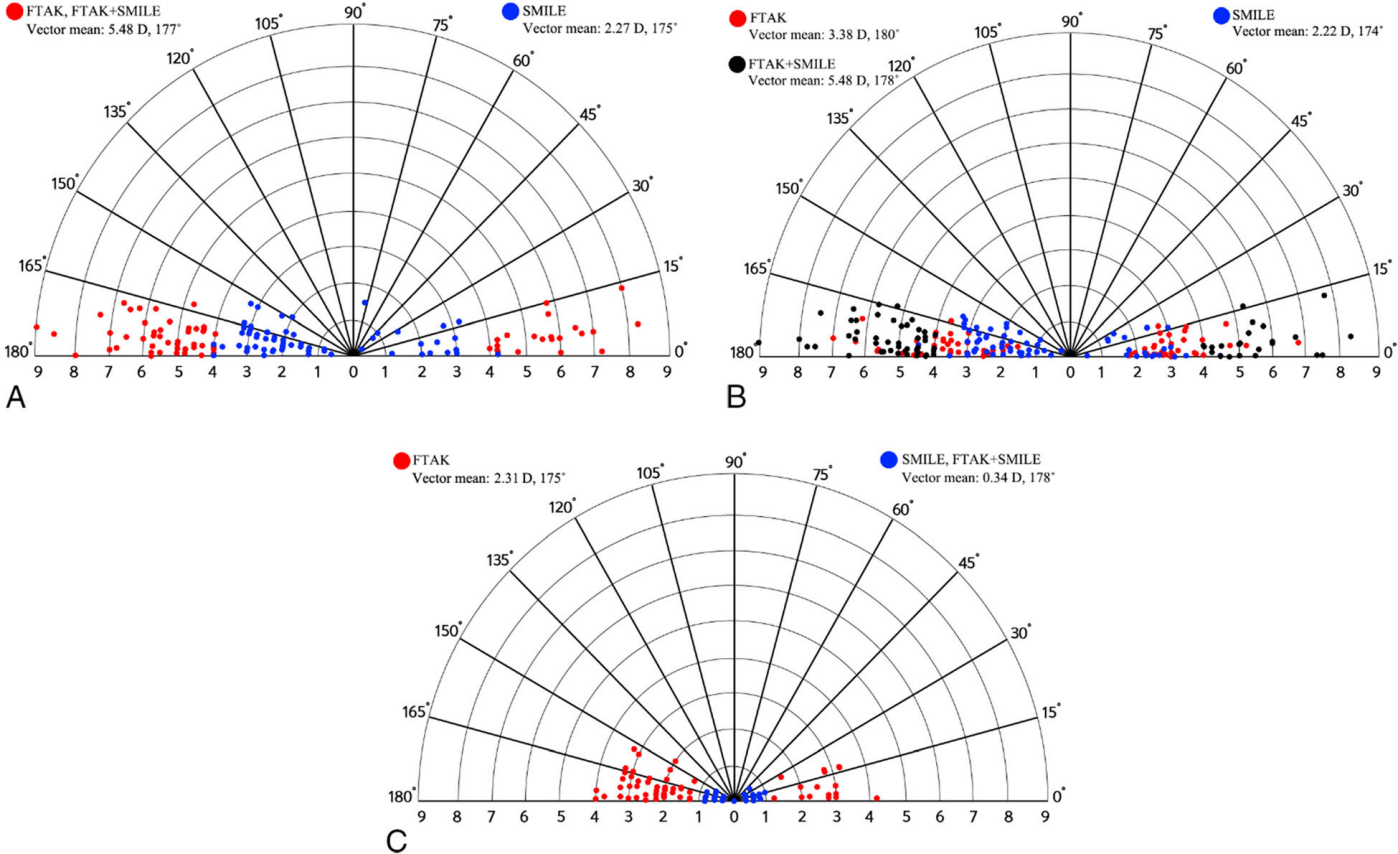
Single-angle polar plots of (**a**) target induced astigmatism (TIA), (**b**) surgically-induced astigmatism (SIA), and (**c**) difference vector (DV) at 1 month after full-thickness astigmatic keratotomy (FTAK) and 24 months after small-incision lenticule extraction (SMILE)

Table 4 shows the vector analysis results for each procedure when used individually or combined. Single-angle polar plots with a mean TIA, SIA, and DV vector for FTAK, SMILE, and the two procedures combined are shown in Fig. 3. Because the target refraction was emmetropia, the TIA represented astigmatism before the procedure and the DV represented the residual astigmatism after each procedure. The mean DV results were 2.27 D × 175° after FTAK and 0.34 D × 178° after both SMILE and the two procedures combined, indicating 2.27 D undercorrection after FTAK and 0.34 D undercorrection after both SMILE and the two procedures combined.

**Table 4:** Vector parameters for full-thickness astigmatic keratotomy (FTAK), small-incision lenticule extraction (SMILE), and the two procedures combined

	FTAK	SMILE	FTAK + SMILE	*p* value
TIA	5.48 ± 1.17	2.27 ± 0.97	5.48 ± 1.17	< 0.001
SIA	3.38 ± 1.18	2.22 ± 0.84	5.39 ± 1.20	< 0.001
DV	2.27 ± 0.97	0.34 ± 0.26	0.34 ± 0.26	< 0.001
ME	−2.10 ± 1.02	− 0.04 ± 0.55	−0.09 ± 0.36	< 0.001
AE	2.08 ± 5.47	− 0.67 ± 4.67	−0.12 ± 1.14	< 0.001
CI	0.63 ± 0.17	0.90 ± 0.40	0.98 ± 0.06	< 0.001
IS	0.41 ± 0.17	0.05 ± 0.33	0.06 ± 0.05	< 0.001

*TIA* target-induced astigmatism, *SIA* surgically-induced astigmatism, *DV* difference vector, *ME* magnitude of error, *AE* angle of error, *CI* correction index, *IS* index of success

*p* value with one-way ANOVA

**Fig. 4:**
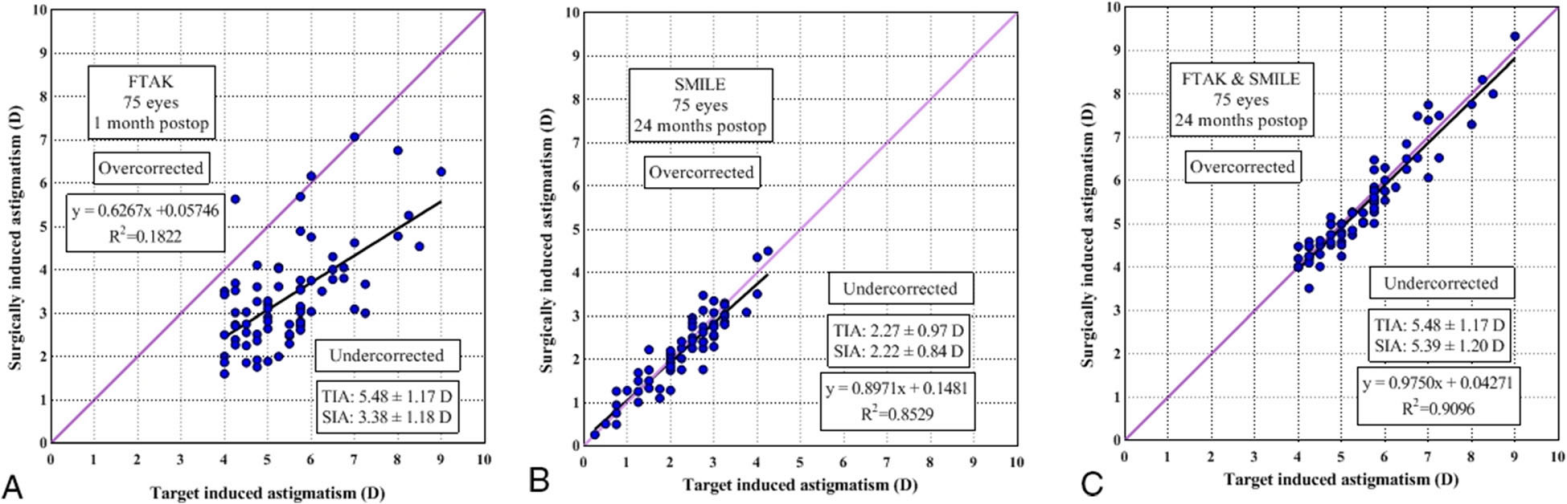
Scatterplot of the target induced astigmatism (TIA) versus surgically-induced astigmatism (SIA) of (**a**) full-thickness astigmatic keratotomy (FTAK), (**b**) small-incision lenticule extraction (SMILE), and (**c**) the two procedures combined

The SIA results were 3.38 ± 1.18, 2.22 ± 0.84, and 5.39 ± 1.20 after FTAK, SMILE, and the two procedures combined, respectively; this difference was statistically significant (*p* < 0.001). The mean AE values were 2.08 ± 5.47, − 0.67 ± 4.67, and − 0.12 ± 1.14 after FTAK, SMILE, and the two procedures combined, respectively; all values were significantly different from zero (*p* < 0.001), indicating that the achieved correction was located in the clockwise direction to the intended axis for SMILE and in the two procedures combined, and in the counterclockwise direction to the intended axis for FTAK. The mean ME was − 2.10 ± 1.02, − 0.04 ± 0.55, and − 0.09 ± 0.36 after FTAK, SMILE, and the two procedures combined; all values were negative, indicating undercorrection. The CI results were all under 1, indicating undercorrection of astigmatism after each procedure and their combination. However, the CI for the two procedures combined was 0.98 ± 0.06, which was significantly high compared to the CI of both FTAK and SMILE individually (*p* < 0.001). The regression line between TIA and SIA had a slope of 0.627, 0.897, and 0.975, indicating 37.3, 10.3, and 2.5% of astigmatism undercorrection after FTAK, SMILE, and the two procedures combined, respectively (Fig. 4).

**Fig. 5:**
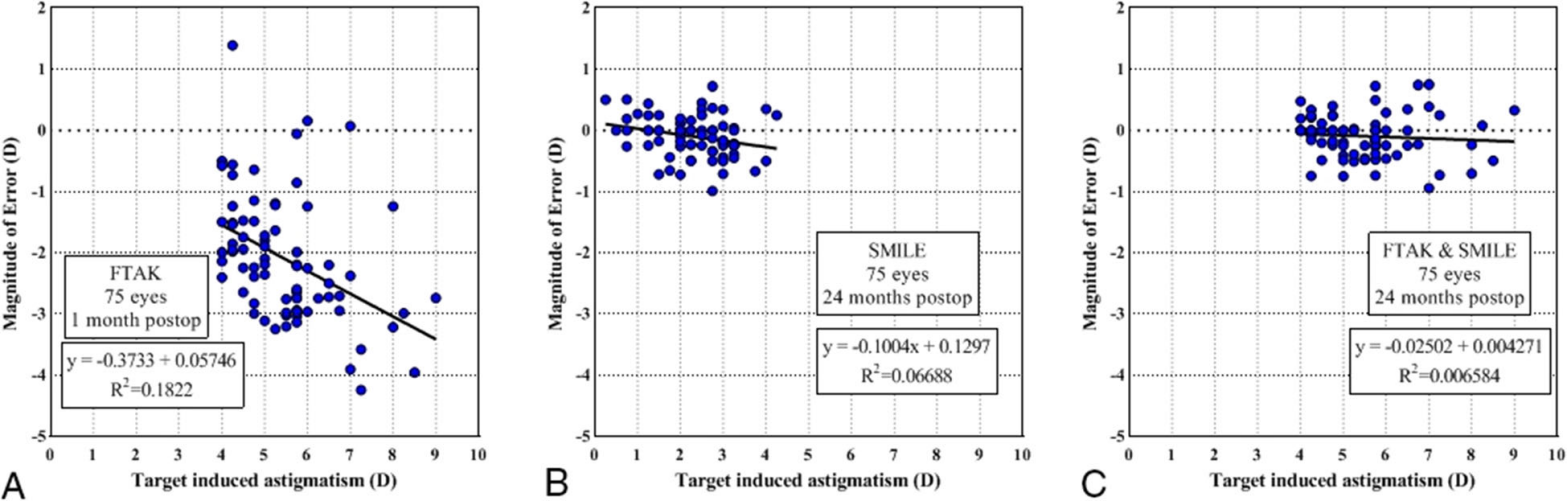
Magnitude of error (ME) versus target induced astigmatism (TIA) after (**a**) full-thickness astigmatic keratotomy (FTAK), (**b**) small-incision lenticule extraction (SMILE), and (**c**) the two procedures combined

A tendency towards increased undercorrection with a high level of TIA can be seen in Fig. 5. This observation was statistically significant after individual FTAK and SMILE procedures (both *p* < 0.001), but was not statistically significant after the two procedures combined (*p* = 0.489).

### Safety and complications

There were no intraoperative or postoperative complications such as of wound dehiscence, infection, epithelial ingrowth, or ectasia during the follow-up period. Also, there was no significant change in the endothelial cell number (*p* = 0.272).

## Discussion

The reasons for the failure to fully correct astigmatism when using SMILE are not fully understood. There have been several possible causes of astigmatic undercorrection after SMILE suggested by many authors. Because of the lack of an active eye tracker, the centration of treatment in SMILE is purely subjective and may vary greatly between surgeons. In addition, there is no standardized method to determine the center of the optic zone, which can be the pupil center or the corneal vertex. Yu et al. [10] found that the decentration was 0.27 ± 0.09 mm from the corneal vertex, and Lazaridis et al. [11] reported a mean decentration of 0.31 ± 0.21 mm after SMILE. However, laser in situ keratomileusis (LASIK) procedures that harbor an active eye tracker also cause decentration of ablation. Some authors reported no significant difference in decentration between LASIK and SMILE [11, 12], and Chan et al. [12] found no significant association between decentration and astigmatic correction after LASIK and SMILE. Cyclotorsion of the eye from standing to supine position is another possible cause of astigmatic undercorrecion after refractive surgery [13]. Moreover, because Visumax does not harbor a cyclotorsion compensation system, cyclotorsion is one of the biggest concerns when treating eyes with high astigmatism through SMILE. Some authors reported axis misalignment after SMILE and the superiority of LASIK in correcting low-to-moderate astigmatism, which was explained by the lack of a cyclotorsion compensation system in SMILE [4, 14, 15]. To compensate for cyclotorsion, manual compensation using corneal marking in the sitting position and rotation of the con after docking was suggested during SMILE, with some reports of better astigmatic correction [16, 17]. However, according to an observation reported by Ganesh et al. [16], 86% of eyes with a high cylinder demonstrated 5 degrees or less of cyclotorsion with none having 10 or more degrees, and the manual marking method could inherently introduce inconsistency ranging from 3.8 to 6.0 degrees. Moreover, a recent study yielded comparable results between SMILE and LASIK when treating over 3.0 D of astigmatism without the use of the manual corneal marking method during SMILE [18]. Besides, there are other factors that may influence astigmatic correction by SMILE, including ocular residual astigmatism, angle kappa, anterior corneal curvature, preoperative axis of astigmatism, and the technique of lenticule extraction [12, 19–22]. Several adjustments of current treatment nomograms have been suggested, leading to a 10% increment in the magnitude of astigmatism correction [4, 23]. Currently, however, there are no standardized nomograms for astigmatism correction.

Although many causes have been suggested for astigmatic undercorrection after SMILE and methods have been proposed to remediate them, none of them has been proved to be true or effective. However, there is a clear tendency that the greater the preoperative astigmatism, the higher the degree of undercorrection [4–6]. Ivarsen and Hjordtal [6] thus reported that an undercorrection of 13% per diopter of attempted correction was observed after treatment of low astigmatism, compared to 16% after treatment of high astigmatism after SMILE. Furthermore, Pederson et al. [4] found that 94 and 63% of patients with 0.5 to 1.0 D and 3.0 to 4.0 D preoperative astigmatism had an ME of less than 0.5 D, respectively.

In the current study, we focused on refraction. Preoperative high astigmatism is one of the biggest risk factors for astigmatic undercorrection, and high astigmatic cornea can cause decentration during suction because of the discrepancy between the corneal curvature and the contact glass surface during SMILE [12]. We thus performed FTAK prior to SMILE to reduce the amount of astigmatism, and hence expect better predictability of SMILE. The steep cornea was flattened by FTAK, which was expected to be helpful in centrating during suction. Furthermore, we also aimed to reduce the lenticule thickness by performing FTAK, thereby reducing astigmatism without altering the spherical equivalent [24]. Moreover, because currently, SMILE is limited to a refractive correction of up to 5.0 D for astigmatism, we could make SMILE applicable to patients who had more than 5.0 D astigmatism preoperatively. We previously reported good efficacy and predictability for the combination of FTAK and SMILE in patients with high astigmatism [7]. However, the follow-up period was of only 6 months, the number of eyes that were included were 13, and no vector analysis was conducted. Herein, we evaluated the effectiveness of astigmatic correction by FTAK and SMILE, both individually and combined, in patients with over 4.0 D astigmatism preoperatively using Alpin’s vector method during a 2-year follow-up period.

The current study demonstrated that when combined, FTAK and SMILE procedures yielded good efficacy, safety, and predictability in correcting eyes with high astigmatism. In our study, an average undercorrection of DV of 0.34 D × 178° was found 24 months after the combined FTAK and SMILE procedures. Pederson et al. [4] reported an average undercorrection of DV of 0.31 D × 91° for eyes with a mean astigmatism of 1.81 ± 1.0 (range: 0.75 to 4.0 D) at 1 year after SMILE. Considering that the preoperative mean astigmatism in our study was 5.48 ± 1.17 D (range: 4.0 to 9.0 D) and 54.7% of eyes had over 5.0 D of astigmatism preoperatively, and were thus inoperable by SMILE alone, the combined FTAK and SMILE procedures showed good effectiveness of astigmatic correction. According to prior studies, the amount of undercorrection after SMILE tends to increase with higher preoperative astigmatism [4–6]. In our study, a tendency toward increased undercorrection with a high level of preoperative astigmatism was not statistically significant after FTAK and SMILE combined (*p* = 0.489), whereas it was statistically significant after FTAK and SMILE individually (both *p* < 0.001). We suggest that this was because the astigmatism was reduced after FTAK, so the TIA of SMILE was relatively low and the predictability of SMILE was increased. Furthermore, 79% of eyes achieved a UDVA of 20/20 or better, 96% of eyes had a CDVA that was equal to or better than preoperative CDVA, and the postoperative SE was − 0.36 ± 0.42 D at 24 months after the combined procedures. No complication was observed during the 2-year follow-up period.

Astigmatic keratotomy (AK) is an effective procedure for the correction of naturally-occurring astigmatism as well as any residual astigmatism in patients who underwent LASIK, lensectomy, or keratoplasty [25–27]. However, the predictability of AK is poor, especially when correcting higher astigmatism [26]. Recently, many authors reported better results using femtosecond laser technology with AK [28–30]. Loriaut et al. [28] reported a mean CI of 0.9 of femtosecond-assisted AK in post-keratoplasty astigmatism, but 50% of eyes were overcorrected. Although the CI of FTAK was 0.63 in our study, only 3 eyes (4%) were overcorrected with FTAK, including 2 eyes that were overcorrected by less than 0.5 D (Fig. 4). That was because we did not have 1 CI as our target for FTAK. Rather, we performed a FTAK procedure to reduce astigmatism so that the accuracy of astigmatic correction with SMILE could be improved. In our study, the mean safety index was 1.02 ± 0.10 after FTAK, which was lower than the safety indices of 1.59, 1.29 reported by Loriaut et al. [28] and Fadlallah et al. [29], respectively. We suggest that this is because these two previous studies performed AK on post-keratoplasty eyes whereas virgin eyes were used in our study, and preoperative CDVAs were 0.5 ± 0.3 and 0.51 ± 0.26 logMAR in Loriaut et al. [28] and Fadlallah et al. [29]’s studies, respectively, which was lower compared to − 0.03 ± 0.07 logMAR in our preoperative CDVA. Even though we used manual incision, full-thickness incision with a uniform keratome was used to avoid inaccuracies in incision depth and length, and we achieved satisfactory results in astigmatism reduction with FTAK, with a SIA of 3.38 ± 1.18 D at 1 month postoperatively. Importantly, we did not note any complications such as wound leak, endophthalmitis, or retinal detachment after FTAK during the follow-up period. It is thought that the long tunnel incision helped prevent not only regression of astigmatism but also wound leakage.

There may be some concerns about refraction change after FTAK as a result from corneal healing [25, 29]. Some authors reported postoperative increase in astigmatism after partial thickness AK on post-keratoplasty eyes, but we performed full-thickness incision, which is preventive of astigmatic regression, on eyes with naturally-occurring astigmatism. Furthermore, there was no significant change in postoperative astigmatism 1 month after FTAK in our previous studies about FTAK [7, 31]. All eyes, in our study, had a stable refraction at 4 weeks after FTAK, so all SMILE procedures were performed within 4 ~ 6 weeks after FTAK. Finally, inaccuracy in astigmatic correction of astigmatic keratotomy may also be a big concern. However, in our study, because residual astigmatism was subsequently corrected with SMILE, any potential inaccuracy did not pose any problems.

In this study, at 1 month after FTAK, SE was not changed compared to preoperative SE (*p* = 0.357). And the mean DV of FTAK was 2.27 ± 0.97 D, which may also be considered as the TIA for SMILE. The amount of astigmatic correction by SMILE was comparable with the results of other studies [4–6]. In the current study, an undercorrection of 10.3% was observed after SMILE, and there was a tendency towards increased undercorrection with a high level of TIA. We suggest that this is because the astigmatism range was 0.25 to 4.25 after FTAK, which was comparable to previous studies, and FTAK was done on the peripheral corneal zone, thereby saving the optic zone.

Two limitations of this study were the lack of evaluation of the causes underlying astigmatic undercorrection and its retrospective design. A prospective study examining the relationship between decentration and the amount of astigmatic undercorrection or a comparative study evaluating the efficacy of SMILE alone versus SMILE combined with FTAK in eyes with 4.0 ~ 5.0 D are thus warranted to corroborate our findings.

## Conclusions

The current study found that combination FTAK and SMILE showed good and stable clinical outcomes during two-year follow-up for the treatment of high astigmatism.

## Data Availability

All data generated or analyzed during this study are available from the corresponding author on reasonable request.

## Abbreviations

SMILE: Small-incision lenticule extraction
FTAK: Full-thickness astigmatic keratotomy
CDVA: Corrected distance visual acuity
UDVA: Uncorrected distance visual acuity
logMAR: Logarithms of the minimum angle of resolution
TIA: Target induced astigmatism
SIA: Surgically induced astigmatism
DV: Difference vector
ME: Magnitude of error
AE: Angle of error
CI: Correction index
IS: Index of success
